# An Antioxidant Phytotherapy to Rescue Neuronal Oxidative Stress

**DOI:** 10.1093/ecam/nen053

**Published:** 2011-02-14

**Authors:** Zhihong Lin, Danni Zhu, Yongqing Yan, Boyang Yu, Qiujuan Wang, Pingniang Shen, Kefeng Ruan

**Affiliations:** ^1^Department of Chinese Medicinal Prescription, China Pharmaceutical University, 639 Longmian Avenue, Jiangning University City, Nanjing, Jiangsu 211198, China; ^2^Department of Pharmacognosy, China Pharmaceutical University, Nanjing, Jiangsu 211198, China; ^3^Department of Physiology, China Pharmaceutical University, Nanjing, Jiangsu 211198, China; ^4^National Engineering Research Center for Traditional Chinese Medicine, Shanghai 201203, China

## Abstract

Oxidative stress is involved in the pathogenesis of ischemic neuronal injury. A Chinese herbal formula composed of *Poria cocos* (Chinese name: *Fu Ling*), *Atractylodes macrocephala* (Chinese name: *Bai Zhu*) and *Angelica sinensis* (Chinese names: *Danggui, Dong quai, Donggui*; Korean name: *Danggwi*) (FBD), has been proved to be beneficial in the treatment of cerebral ischemia/reperfusion (I/R).This study was carried out to evaluate the protective effect of FBD against neuronal oxidative stress *in vivo* and *in vitro*. Rat I/R were established by middle cerebral artery occlusion (MCAO) for 1 h, followed by 24 h reperfusion. MCAO led to significant depletion in superoxide dismutase and glutathione and rise in lipid peroxidation (LPO) and nitric oxide in brain. The neurological deficit and brain infarction were also significantly elevated by MCAO as compared with sham-operated group. All the brain oxidative stress and damage were significantly attenuated by 7 days pretreatment with the aqueous extract of FBD (250 mg kg^−1^, p.o.). Moreover, cerebrospinal fluid sampled from FBD-pretreated rats protected PC12 cells against oxidative insult induced by 0.2 mM hydrogen peroxide, in a concentration and time-dependent manner (IC_50_ 10.6%, ET_50_ 1.2 h). However, aqueous extract of FBD just slightly scavenged superoxide anion radical generated in xanthine–xanthine oxidase system (IC_50_ 2.4 mg ml^−1^) and hydroxyl radical generated in Fenton reaction system (IC_50_ 3.6 mg ml^−1^). In conclusion, FBD was a distinct antioxidant phytotherapy to rescue neuronal oxidative stress, through blocking LPO, restoring endogenous antioxidant system, but not scavenging free radicals.

## 1. Introduction

Acute ischemic stroke is a leading cause of death in the majority of countries [1]. Evidence affords the involvement of oxidative stress in neuronal injury during brain ischemia/reperfusion (I/R) [2–4]. The lethal process was accompanied by elevated free radicals, including superoxide anion (O_2_^•−^), hydroxyl radical (^•^OH) and hydrogen peroxide (H_2_O_2_), as well as progressive depletion in endogenous antioxidant system, including antioxidant enzymes, superoxide dismutase (SOD), glutathione peroxidase (GSH-Px) and catalase, or antioxidants, glutathione (GSH), Vitamin (Vit) C and Vit E (*α*-tocopherol) [5]. Pathological free radicals directly damage neuronal proteins, lipids and DNA; generate toxic lipid peroxides and ultimately contribute to brain infarction and neurobehavioral symptoms. Although free radical scavengers, for example, edaravone [6] or extract of *Ginkgo biloba* (EGb761) [7], have been demonstrated to be antagonistic to brain I/R, the anti-I/R agents available were still far from sufficient [8].

In traditional Chinese medicine, *Poria cocos* (Chinese name: *Fu Ling*), the dried sclerotia of *P. cocos* (Schw.) Wolf (Polyporaceae), is used as a diuretic, sedative and tonic [9]. Triterpene acids and polysaccharides are the principal ingredients of *P. cocos* that are responsible for diverse bioactivities, including antitumor, anti-inflammatory, nematicidal, antioxidant, anti-rejection and anti-emetic effects, and act as inhibitors against DNA topoisomerases, phospholipase A2 and cholinesterase (see [9–16], Table 1). The dried rhizome of *Atractylodes macrocephala* Koidz. (Compositae) (Chinese name: *Bai Zhu*) is used as a digestive and a tonic, in which volatile oils, polysaccharides, sesquiterpenes and flavonoids were identified with anti-inflammatory, hypoglycemic and gastrointestinal inhibitory effects, and so forth. (see [10–12, 17–20], Table 1). The dried root of *Angelica sinensis* (Oliv.) Diels (Umbelliferae) (Chinese names: *Danggui, Dong quai, Donggui*; Korean name: *Danggwi*) is used as a vital blood tonic and especially to treat gynecological diseases. Due to its varied constituents, such as volatile oils, polysaccharides and coumarin derivatives, several pharmacological actions may be attributed to Danggui, including anticoagulation and antiplatelet activities, as well as hematopoiesis, immune support and uterine tonicity (see [12, 13, 21–33], Table 1). These three essential herbs have been used for thousands of years in Asia and first documented in *Shen-nong-ben- cao-jing*, the first Chinese medical pharmacopoeia written in the Han dynasty.

The beneficial effects of these plants on cerebrovascular disorders have drawn increasing attention in recent research. Clinically, a great deal of traditional herbal formulae comprising *Fu Ling, Bai Zhu* and *Danggui* (FBD) were applied to cure ischemia stroke and vascular dementia (VD), mostly with good efficacy. Statistical analysis showed that the three herbs are frequently used in formulae, notably anti-stroke/VD formula *Toki-Shakuyaku-San* or *Yi-Gan San* [34, 35]. To some extent, clinical neuroprotection of the three herbs was shown to be relevant to their antioxidant properties [10, 36, 37]. As traditional Chinese nourishing-tonifying drugs, crude extracts of Fu Ling, Bai Zhu and/or Danggui have the capacity to inhibit cellular lipid peroxidation (LPO) induced by free radicals, for example, H_2_O_2_ [21–23], as well as preserve tissue GSH status and GSH-Px activity [11, 24]. However, *in vitro*, their direct free radical scavenging activities are relatively weak due to high concentration in various biochemical reactions, including xanthine-xanthine oxidase (XO) system and Fenton reaction system [12, 13]. Therefore, it is hypothesized that FBD exerts its protective effects against I/R-induced neuronal oxidative stress, largely via inhibiting LPO and maintaining endogenous antioxidant system, instead of scavenging free radicals.

The primary aim of this study was to evaluate the herbal formula on neuronal oxidative stress induced by middle cerebral artery occlusion (MCAO) *in vivo* and by H_2_O_2_ *in vitro*. In addition, we evaluated scavenging activities of FBD against O_2_^•−^ generated in xanthine-XO system and ^•^OH generated in Fenton reaction system to assess its antioxidant properties.

## 2. Methods

### 2.1. Preparation of Aqueous Extract of FBD

The three herbal materials used in this work were purchased from Nanjing herbal materials company (Nanjing, China) and authenticated by Prof. Boyang Yu, Department of Pharmacognosy, China Pharmaceutical University. Clinically, a single formula of FBD consisted of 10 g *P. cocos*, 5 g *A. macrocephala* and 3 g *A. sinensis* and the aqueous extract of FBD was prepared the three components were macerated for 30 min, decocted for 30 min with 8 times (v/w) double-distilled H_2_O and the filtrate obtained was concentrated and dried in vacuum at 60°C into a brown powder, with a yield of 12.5% (w/w).

### 2.2. Reagents and Chemicals

Vit E, Vit C, 2,3,5-triphenyltertrazolium chloride (TTC), 1,10-phenanthroline and 3-(4,5-dimethylthiazol-2-yl)-2,5-diphenyltetrazolium bromide (MTT) were purchased from Sigma (St Louis, MO). EGb761 was purchased from Schwarz Pharma AG (Monheim, Germany). Medical kits for malondialdehyde (MDA), nitric oxide (NO), GSH and SOD assays were purchased from Nanjing Jiancheng Bioengineering Institute (Nanjing, China).

### 2.3. Animals and Pretreatment

Male Sprague-Dawley rats weighting 250–350 g were randomized into four groups: rats in FBD-pretreated groups received FBD (250 mg kg^−1^, p.o.), while EGb761-pretreated rats were given EGb761 (24 mg kg^−1^, p.o.), as positive control. Sham-operated group and vehicle-pretreated group were given p.o. vehicle 0.5% carboxymenthylcellulose-saline. Vehicle or drugs were administrated once daily for 7 consecutive days. The animal handling procedures were in compliance with the China National Institutes of Healthy Guidelines for the Care and Use of Laboratory Animals.

### 2.4. Middle Cerebral Artery Occlusion

One hour after the seventh administration, rats were subject to 1 h right MCAO using the intraluminal filament technique [38]. Briefly, rats were anesthetized with chloral hydrate (400 mg kg^−1^, i.p.). The right common carotid artery was exposed at the level of the external and internal carotid artery (ECA and ICA) bifurcation. A 4-0 monofilament nylon suture was inserted into the ECA and advanced into the ICA for 17–20 mm until a slight resistance was felt, to block the origin of the middle cerebral artery. One hour after MCAO, the suture was slowly withdrawn. The sham-operated rats did not suffer MCAO, except with exposure to ECA and ICA. Animals were then returned to their cages for 24 h and closely monitored, with body temperature kept at 37 ± 0.5°C.

### 2.5. Neurological and Histological Examination

The neurological deficits in rats were assessed after 24 h reperfusion. Ten rats from each group were assigned a numerical score on a 5-point scale as described: no neurological deficit = 0; failure to fully extend right paw = 1; circling to right = 2; falling to right = 3; did not walk spontaneously and had depressed levels of consciousness = 4 [39]. Then, rats were killed and brain tissue was removed and sliced into 2.0 mm thick coronal sections. Brain slices were incubated in 2% TTC saline solution at 37°C for 30 min, then fixed in 10% phosphate-buffered formalin for 45 min. Infarct volume in brain slices, outlined in white, were captured with a digital camera and measured by image analysis system (Zeiss AxioVs 40, Oberkochen, Germany) and calculated using the following equation: % infarct volume = infarct volume/slice volume × 100%.

### 2.6. Neurochemical Assays

Twenty-four hours after reperfusion, rats were sacrificed and cortical cortexes were collected. A 10% (w/v) homogenate was prepared in ice-cold saline and the supernatant was obtained after centrifugation at 3000 r.p.m. for 15 min. Neurochemical assays were conducted in accordance with the specification of medical kits. When unsaturated fatty acids undergo LPO, MDA is formed. Thiobarbituric acid reaction was used to determine MDA (expressed as *μ*mol g^−1^ protein) [40]. Nitrite in cortical supernatant was measured after reaction with Griess reagent (sulfanilamide 1%, naphthylethylene diamine 0.01%, H_3_PO_4_ 5%) with sodium nitrite as a standard, by which NO production might be assessed as micromole per gram of protein [41]. The assay for SOD was based on its ability to inhibit the oxidation of oxymine by the xanthine-XO system (expressed as U mg^−1^ protein) [42]. GSH (expressed as *μ*mol g^−1^ protein) was measured through a reaction using dithiobisnitrobenzoic acid, as described by Ball [43]. Protein concentration was measured by Lowry method with bovine serum albumin as standard.

### 2.7. Sampling of Cerebrospinal Fluid

Fresh cerebrospinal fluid (CSF) was sampled 0, 0.5, 1, 1.5, 2.0 and 2.5 h after the seventh administration, from FBD-pretreated rats free of MCAO, using three rats per time point. In short, rats were anesthetized with chloral hydrate (400 mg kg^−1^, i.p.), 30–50 *μ*l CSF was carefully pricked from bulbus medullae pool using a sterile injection syringe [44]. After centrifugation at 3000 r.p.m. for 10 min, the CSF containing FBD (CSF-FBD) was stored at −20°C.

### 2.8. Oxidative Insult in PC12 Cells Induced by H_2_O_2_

Neuron-like pheochromocytoma (PC12) cells were provided by Institute of Cells Biology (Shanghai, China). The cells were suspended in Dulbecco Modified Eagle's Medium supplemented with 10% heat-inactivated newborn calf serum, benzylpenicillin (100 kU l^−1^) and streptomycin (100 mg l^−1^) and incubated at 37°C in 5% CO_2_. PC12 cells were exposed to H_2_O_2_ (200 *μ*M) for 1 h to induce oxidative insult, then treated with CSF-FBD (v/v), Vit E (10 *μ*M) or blank CSF. Twenty hours later, MTT assay was performed to observe the cell viability in PC12 cells [37]. Briefly, MTT solution (0.5 mg ml^−1^) was added to each culture well. After incubation for 4 h, the formazan crystals were dissolved by addition of 50 *μ*l dimethyl sulfoxide and read at dual wavelength, 570 nm/650 nm.

### 2.9. Superoxide Radical Generated in Xanthine-Xanthine Oxidase System

According to Link and Riley [45], the xanthine-XO system of a final volume of 2.0 ml contained 375 *μ*mol l^−1^ xanthine, 6.25 U l^−1^ XO, 500 *μ*mol l^−1^ hydroxylamine, 100 mmol l^−1^ Na_2_HPO_4_ *·*12H_2_O–NaH_2_PO_4_ *·*2H_2_O buffer (pH 7.8) and FBD or Vit C at different concentrations (0.05–5 mg ml^−1^). Reaction was initiated by adding XO and the tubes were incubated at 37°C for 40 min and then terminated by placing in an ice bath. The absorbance of nitrate from hydroxylamine was measured at 550 nm after reaction with Griess reagent. O_2_^•−^ scavenging by FBD was calculated by the following equation: %Scavenging rate = [1−*A*_1_/*A*_0_] × 100% [*A*_0_: Control; *A*_1_: Drug].

### 2.10. Hydroxyl Radical Generated in Fenton Reaction System

The Fenton reaction system of a final volume of 2 ml contained 0.75 mmol l^−1^ FeSO_4_, 0.75 mmol l^−1^ 1,10-phenanthroline, 0.8 mmol l^−1^ H_2_O_2_, 150 mmol l^−1^ PBS buffer and FBD or Vit C at different concentrations (0.05–5 mg ml^−1^). Reaction was initiated by adding H_2_O_2_ and the tubes were incubated for 60 min in a water bath at 37°C. The absorbance of the Fe^2+^-phenanthroline complex was measured at 510 nm [46]. All values represent the average of triplicate experiments. ^•^OH scavenging by FBD was calculated by the following equation: %Scavenging rate = [1−(*A*_2_−*A*_1_)/(*A*_2_−*A*_0_)] × 100% [*A*_0_: Control; *A*_1_: Drug; *A*_2_: Blank without drug and H_2_O_2_].

### 2.11. Statistical Analysis

SPSS 12.0 software and Origin 7.0 software were applied to analyze experimental data and results were expressed as mean ± SD. All data were evaluated with analysis of variance (ANOVA) following by Dunnett's *t*-test for multiple comparisons and *P* < .05 indicates that the difference was statistically significant.

## 3. Results

### 3.1. Neuroprotective Effects of FBD In Vivo

**Figure 1:**
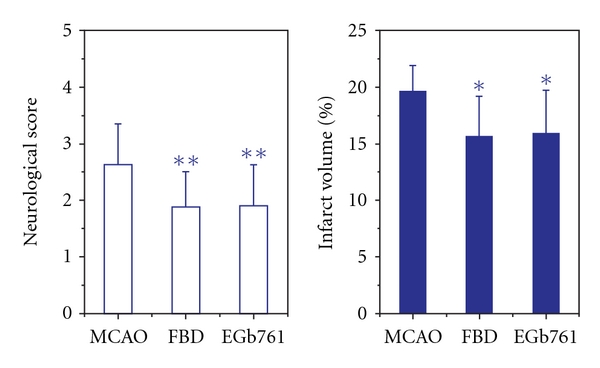
Effects of aqueous extract of FBD on neurological score and brain infarction in rats subject to MCAO. Each column represents the mean ± SD of 10–12 rats. Significance was evaluated with one-way ANOVA following by two-sided Dunnett's *t*-test. _*_ *P* < .05, _**_ *P* < .01 versus the vehicle-pretreated MCAO group.

Rats surviving more than 24 h awakened from anesthesia with a moderately severe left hemiparesis and circling movements. TTC staining indicated that infarction zone existed in right lobus temporalis cortical and striatal tissues. The neurological score and infarct size in the vehicle-pretreated MCAO rats rose up to 2.6 ± 0.7 and 19.7%  ± 2.2%, respectively, indicating that I/R resulted in neuronal injury. In comparison to the vehicle-pretreated group, FBD (250 mg kg^−1^) significantly reduced the neurological score by 28.4% (*P* < .05) and infarct size by 20.1% (*P* < .01). Its actions were to some extent stronger than those of 24 mg kg^−1^ EGb761 (by 27.6%, *P* < .05 and by 18.9%, *P* < .01, resp., Figure 1).

### 3.2. Antioxidant Effects of FBD In Vivo

MCAO-induced neurochemical changes are shown in Table 2. After 24 h reperfusion, MDA and NO contents in vehicle-pretreated group rose significantly (*P* < .01); in contrast, GSH content and SOD activity reduced significantly (*P* < .01), which implied that oxidative stress occurred. With respect to the vehicle-pretreated group, FBD (250 mg kg^−1^) significantly reduced MDA and NO production (*P* < .01) and restored SOD activity (*P* < .01) and GSH content (*P* < .05); likewise, EGb761 significantly suppressed oxidative stress to a similar extent.

### 3.3. Antioxidant Activity of FBD Ex Vivo

**Figure 2:**
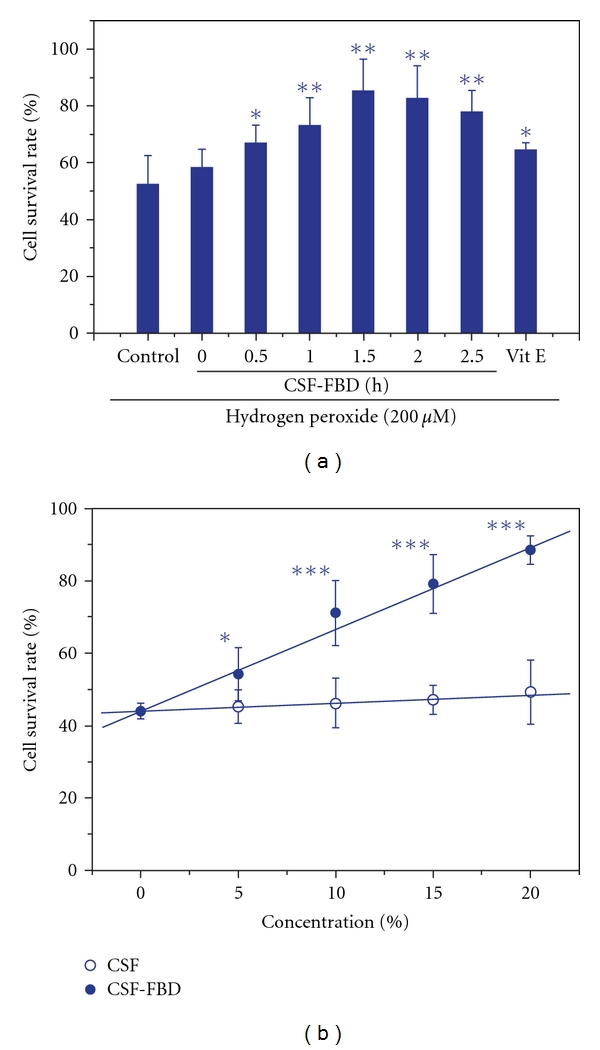
Effect of rat CSF-FBD on PC12 cells induced by hydrogen peroxide. All the data were shown as the mean ± SD, *n* = 6. Significance was evaluated with one-way ANOVA following by two-sided Dunnett's *t*-test. *P < .05, _**_ *P* < .1, _***_ *P* < .001 versus blank CSF.

Incubation with H_2_O_2_ for 3 h significantly reduced cell viability. However, when the cells were treated with rat CSF-FBD, the observed cell toxicity was significantly attenuated. As illustrated in Figure 2, CSF-FBD markedly reduced H_2_O_2_ injury within 1.5 h in a time-dependent manner (ET_50_ 1.2 h) and concentration-dependant manner within 20% (IC_50_ 10.6%). Meanwhile, blank CSF had no obvious influence on the control PC12 cells and Vit E (10 mM) protected PC12 cells by only 25.2%.

### 3.4. Free Radical Scavenging Activity of FBD In Vitro

**Figure 3:**
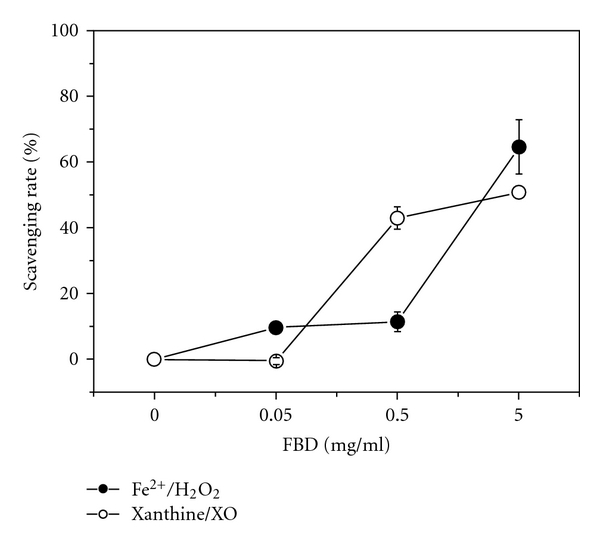
Scavenging activities of aqueous extract of FBD against superoxide anion radical generated in xanthine-XO system and hydroxyl radical generated in Fe^2+^/H_2_O_2_ Fenton reaction system with ascorbic acid as a positive control. Values were means ± SD, *n* = 6.

Direct free radical scavenging activity by FBD is shown in Figure 3. At concentrations of 0.05–5.0 mg ml^−1^, FBD exhibited concentration-dependent scavenging activities against O_2_^•−^ generated in xanthine-XO system and ^•^OH generated in a Fenton reaction system, with IC_50_ 2.4 mg ml^−1^ and 3.6 mg kg^−1^, respectively, higher than those of Vit C (IC_50_ 0.01 mg ml^−1^ and 0.25 mg ml^−1^, resp.).

## 4. Discussion

This study demonstrates the neuroprotective potential of FBD against MCAO-induced oxidative stress in rats, as well as H_2_O_2_-induced oxidative stress in neuron-like PC12 cells. Its neuroprotection appears to reduce LPO and restore endogenous antioxidant system but not scavenge free radicals.

It is well documented that transient focal MCAO results in neurological and histological abnormality. Our results indicated that pretreatment with FBD offered protection against cortical and striatal neuronal damage induced by MCAO, as FBD reduced the neurological score and infarct size (Figure 1), in harmony with other studies [47, 48].

Free radical involvement in the development of I/R-induced brain injury is well investigated [3, 4], among which, O_2_^•−^ and ^•^OH are potent by inducing LPO [49]. The highly reactive ^•^OH is formed from H_2_O_2_ in the presence of divalent metal ions, especially Fe^2+^ and Cu^2+^, via the Fenton reaction. In addition, during ischemia, xanthine dehydrogenase undergoes irreversible proteolytic conversion to XO, producing O_2_^•−^ and H_2_O_2_ in the presence of oxygen [3]. O_2_^•−^ does not directly induce LPO but can react with ^•^NO to form cytotoxic peroxynitrite (ONOO^−^) [50]. We found that focal MCAO induced increases of LPO and NO (Table 2), in agreement with recent studies [51, 52]. FBD inhibited NO production but its scavenging activity against either ^•^OH or O_2_^•−^ was feeble compared to that of Vit C (Figure 3), supporting previous findings that *P. cocos* and *A. sinensis* were relatively weak natural free radical scavengers [13].

The overproduction of free radicals can be detoxified by endogenous antioxidants, causing their cellular stores to be depleted [52, 53]. Physiologically, SOD reacts with O_2_^•−^ to form H_2_O_2_; Catalase and GSH-Px are involved in the detoxification of H_2_O_2_; GSH, which is considered the most prevalent and important intracellular non-protein thiol, has a crucial role as a free radical scavenger. Here, GSH content and SOD activity were significantly reduced (Table 2). Similar to EGb761, FBD significantly prevented SOD activity and GSH content decline caused by MCAO.

In addition to restoring the endogenous antioxidant system, anti-LPO activity was also implicated in the antioxidant properties of FBD. In 1996, Taylor et al. observed the inhibition of T cells by human CSF, and in 2000, Nakagawa et al. found human CSF altered intracellular calcium regulation in endothelial cells [54, 55]. Since CSF is the natural vehicle for CNS agents, both reports enlightened us to design a novel experimental method to evaluate neuroeffectiveness of FBD *ex vivo*. PC12 cells injured by H_2_O_2_ are a typical model used to evaluate anti-LPO activity of drug on neuronal oxidative stress [56, 57]. In this work, CSF-FBD attenuated oxidative insult affects PC12 cells in both time- and concentration-dependent manner (Figure 2), in accordance with *in vivo* finding that MDA level in MCAO-subjected rats was depressed by FBD extract.

The exact mechanism by which FBD abated oxidative stress is not yet clear but it is strongly believed that recently identified active compounds may be responsible. Triterpenes from *Fu Ling* inhibited FeCl_2_-ascorbic acid induced LPO and lysis of red blood cells [14]. Atractylon from *Bai Zhu* inhibited LPO by CCl_4_ in liver lesion and its acetylene compound (6E,12E)-tetradecadiene-8,10-diyne-1,3-diol diacetate suppressed gastric lesions induced by I/R, via inhibition of XO [17, 18]. Z-ligustilide from *Danggui* protected against H_2_O_2_-induced cytotoxicity in PC12 cells and forebrain I/R by enhancing antioxidant defense [25, 26]. Coniferyl ferulate is the main antioxidant from essential oil of *Danggui* [27] and ferulic acid could reduce neuronal damage from exposure to iron, hydroxyl and peroxyl radicals [28, 29]. In addition, *Danggui* polysaccharides protected macrophages against tert-butylhydroperoxide-induced oxidative injury [30, 31].

In conclusion, our present findings suggested that FBD might exert protection against neuronal oxidative stress, induced by either MCAO *in vivo* or H_2_O_2_ *in vitro*. It is a distinct botanical antioxidant agent to reduce LPO and restore the endogenous antioxidant system, without the activity of free radical scavengers. This research expands and elaborates the biological model underlying one complementary and alternative medicine treatment for neuronal oxidative stress.

## Figures and Tables

**Table 1:** Active ingredients and physio-pharmacological functions of *P. cocos*, *A. macrocephala* and *A. sinensis*.

Herbs	Active ingredients	Physio-pharmacological functions
*P. cocos*	Triterpene acids, Polysaccharides	Antitumor activity and DNA topoisomerases inhibitory activity, anti-inflammatory and anti-phospholipase A(2) activity, nematicidal, antioxidant, anti-cholinesterase, anti-rejection, anti-emetic and anti-nephritic effects
*A. macrocephala*	Volatile oils, Polysaccharides, Sesquiterpenes, Flavonoids	Anti-inflammatory, antitumor activity, gastrointestinal inhibitory effect, suppression of allergic diarrhea and uterine contraction, antioxidant and hypoglycemic effects, diuresis angiogenesis
*A. sinensis*	Volatile oils, Polysaccharides, Coumarin derivatives, Organic acids, Vitamins and minerals	Anticoagulation, antiplatelet activity, hematopoiesis, immune support, anti-inflammatory, antioxidant, antifibrotic and antispasmodic effects, uterine tonicity

**Table 2:** Effects of the aqueous extract of FBD on the contents of MDA, NO and GSH, and SOD activity in rat brain subject to MCAO.

Group	Dose (mg/kg)	NO (*μ*mol/g prot)	MDA (*μ*mol/g prot)	GSH (*μ*mol/g prot)	SOD (U/mg prot)
Sham		4.58 ± 0.75	3.77 ± 0.83	45.33 ± 5.54	143.07 ± 26.65
MCAO		7.94 ± 0.74^##^	6.68 ± 0.54^##^	27.08 ± 3.92^##^	96.57 ± 22.78^##^
EGb761	24	6.01 ± 0.96**	4.56 ± 1.72**	35.03 ± 6.06**	124.43 ± 15.77**
FBD	250	6.25 ± 0.80**	4.64 ± 0.96**	32.51 ± 5.00*	131.73 ± 22.40**

All the data were shown as the mean ± SD, n = 10−12. Significance was evaluated with one-way analysis of variance (ANOVA) following by two-sided Dunnett's *t*-test. ^##^ *P* < .01 versus the sham-operated group; *P < .05, **P < .01 versus the vehicle-pretreated MCAO group.
